# Avoidance of Bereavement-Related Stimuli in Chinese Individuals Experiencing Prolonged Grief: Evidence from a Dot-Probe Task

**DOI:** 10.3389/fpsyg.2017.01201

**Published:** 2017-07-17

**Authors:** Meng Yu, Suqin Tang, Chenyi Wang, Zhendong Xiang, Wei Yu, Wei Xu, Jianping Wang, Holly G. Prigerson

**Affiliations:** ^1^Beijing Key Laboratory of Applied Experimental Psychology, National Demonstration Center for Experimental Psychology Education, Faculty of Psychology, Beijing Normal University Beijing, China; ^2^Department of Social Work and Social Administration, The University of Hong Kong Hong Kong, China; ^3^Department of Clinical Psychology, Capital Medical University Beijing, China; ^4^Center for Research on End-of-Life Care, Weill Cornell Medicine College, New York NY, United States

**Keywords:** prolonged grief, bereavement, attentional bias, avoidant tendency, dot-probe task

## Abstract

**Background:** Attentional bias refers to a preference for (e.g., vigilance) or a shifting away (e.g., avoidance) of one’s focus with respect to specific stimuli. Accumulating evidence suggests that an attentional bias toward death/threat-related stimuli exists in bereaved individuals experiencing prolonged grief (PG). Measuring for different processing may reflect different cognitive characteristics. Therefore, this study sought to compare information-processing biases in Chinese individuals with high versus low levels of PG symptomatology at supraliminal and subliminal levels, respectively.

**Method:** A 2 (grief level) × 2 (consciousness level) × 2 (word type) three-factor mixed design with supraliminal and subliminal tasks was utilized in the current study. Based on their Prolonged Grief Questionnaire-13 (PG-13) scores, 38 participants were included in the low-PG group, and 34 individuals were included in the high-PG group. All the participants completed a dot-probe task in which they were primed with death-related and life-related words paired with neutral stimuli.

**Results:** High-PG individuals were slower in reacting to the death-related information in both supraliminal and subliminal tasks. After controlling for other symptoms in the backward deletion regression, PG-13 scores significantly predicted the avoidance tendency to death-related words in the supraliminal task, while anxiety was the best predictor of turning one’s vision away from death-related stimuli in the subliminal trials.

**Conclusion:** The results suggested that high PG is associated with a tendency to avoid death-related words. Future research is needed to explore interventions that address the avoidance of death-related stimuli among individuals with elevated, or diagnosable, levels of PG.

## Introduction

Accumulating evidence has supported the fact that after the death of a loved one, people may go through various grief experiences (Bonanno and Kaltman, 2001; Prigerson et al., 2009). Although most people succeed in recovering from the loss, approximately 10–15% of bereaved people still experience persistent and disabling symptoms; in fact, many people suffer from prolonged grief disorder (PGD) (Bonanno and Kaltman, 2001; Prigerson et al., 2009; He et al., 2014; Bryant et al., 2015; Heeke et al., 2015; Li and Prigerson, 2016). A meta-analysis further revealed a pooled prevalence of PGD of 9.8% (Lundorff et al., 2017). According to the latest proposal for DSM-5 and ICD-11, PGD is defined as experiencing bereavement, yearning (e.g., craving, pining, or longing) for the deceased daily or to a disabling degree, and demonstrating cognitive, emotional, and behavioral symptoms (e.g., difficulty accepting the loss, avoidance of reminders of the reality of the loss) for at least 6 months after the death (Prigerson et al., 2009; Maciejewski et al., 2016; Prigerson and Maciejewski, 2017). Although some bereaved individuals do not meet PGD diagnostic criteria, many still experience elevated but subsyndromal levels of prolonged grief symptoms. The existence of PG (previously known as “complicated grief” [CG]) has been reported in numerous studies.

Numerous empirical studies have demonstrated that PG symptoms are indeed distinct from anxiety, depression, and post-traumatic stress disorder (Prigerson et al., 1995, 1996; Bonanno et al., 2007; Dillen and Fontaine, 2009; Prigerson et al., 2009). The core features of anxiety disorders are excessive fear and anxiety, while the core features of depression are loss of interest and depressive emotion (DSM-5; American Psychiatric Association, 2013). In addition to intrusive memories about death, the essential difference between PTSD and PGD is that individuals with PG may be left with vivid images of what occurred when the deceased lived and avoid reminders of the deceased (Armour, 2007).

Some researchers proposed that PG individuals may show cognitive and behavioral avoidance after bereavement (Boelen et al., 2006). It is postulated that these individuals may avoid threatening clues that could make them confront the reality of the loss. Similarly, people with PG symptoms may demonstrate cognitive avoidance, such as suppressing memories or ruminating about the loss rather than reconstructing the implications of the loss (Boelen et al., 2006). Avoidance has been shown to interfere with the integration of the loss into pre-existing schemas (Maccallum and Bryant, 2010) and is regarded as a crucial component of CG (Shear et al., 2007). The mediating effect of experiential avoidance between several variables and CG symptoms was also investigated. Experiential avoidance may mediate the relationship between grief rumination and CG (Eisma et al., 2013) and mediate the association between suicidal bereavement and CG (Nam, 2016). Therefore, experiential avoidance may contribute to the formation of CG.

Nevertheless, research has revealed discrepant findings, suggesting that vigilance may also appear in CG individuals. Clinical observations revealed that individuals with PG tended to not only avoid clues related to death but are also often easily reminded of the loss (e.g., Shear and Shair, 2005; Boelen et al., 2006; Prigerson et al., 2008), implying a hypervigilance to cues related to the deceased. Vigilance is defined as faster orientation toward emotional stimuli (Koster et al., 2004). Attention to particular stimuli (e.g., vigilance) or shifting attention away from specific stimuli (e.g., avoidance) is referred to as attentional bias (Mogg et al., 2004b; Bullock and Bonanno, 2013). Moreover, empirical studies have demonstrated an attentional bias toward trauma-related/threatening information in CG individuals. Bullock and Bonanno (2013) reported that when primed with emotional stimuli by a dot-probe task, individuals suffering from CG attended away from sad faces when they were subliminally primed with their deceased spouse’s name, highlighting the existence of avoidance tendency in CG participants. By conducting a modified emotional Stroop task, Maccallum and Bryant (2010) observed that CG participants had a tendency to prefer death-related words compared to bereaved individuals without CG, indicating a vigilant inclination to the clues associated with death. In addition, Eisma et al. (2015) used an approach-avoidance task to assess the behavioral tendencies in PG individuals. This study demonstrated the existence of an avoidance tendency to loss-related stimuli during 1500–10000 ms in high ruminators experiencing bereavement, which confirmed no vigilance inclination and subsequent disengagement in the first 1500 ms. However, Maccallum et al. (2015) used a modified approach-avoidance task and showed that approaching and avoidance co-existed in PG participants, which was inconsistent with the findings of the previous study.

Previous research has explored the features of information processing of PG individuals and discovered two stages: automatic and strategic processing (see review; Cisler and Koster, 2010). Generally, automatic processing refers to processing that induces free and unintended reactions without control or awareness, namely, at the subliminal level, whereas strategic processing refers to processing that is controllable or conscious (Cisler and Koster, 2010). Research regarding anxiety and depression has examined the roles of these types of processing. For instance, several researchers have demonstrated that when primed with a subliminal duration (e.g., 100 ms), anxious individuals reacted faster to threat-related stimuli, suggesting a preferential or vigilant tendency (Koster et al., 2006a,b). However, at a longer stimulus duration (e.g., 1250 ms), anxious participants demonstrated attentional avoidance toward threat cues (Mogg et al., 2004a; Koster et al., 2005). Hence, measuring for different processing could reflect different cognitive characteristics. Nevertheless, individuals experiencing PG are characterized by avoiding information associated with the loss, while seeking clues related to the deceased (Shear and Shair, 2005; Boelen et al., 2006; Prigerson et al., 2008). Therefore, one of the objectives of the current study was to explore the cognitive features at different stages of processing with subliminal and supraliminal tasks, respectively.

Additionally, researchers have increasingly paid attention to the cross-cultural comparison between bereaved individuals from China and western countries, and the results indicated that compared with Western individuals, Chinese individuals experiencing CG were more inclined to present deliberate avoidance (Bonanno et al., 2005) and had higher grief-related functional impairment and higher levels of depressive symptoms (Xiu et al., 2016). In contrast, Burton et al. (2012) showed a consistent consequence across cultures. These authors found that bereaved participants meeting the criteria for CG reported less forward-focused coping strategies and less overall coping flexibility than asymptomatic bereaved and married individuals. Nevertheless, by contrast, there still is a lack of empirical research investigating avoidance or vigilance among Chinese individuals with PGD.

Empirical research exploring the underlying mechanisms of PG in this area remains insufficient and controversial. To clarify the specific processing mechanism, a dot-probe paradigm was adopted in the current study to detect attentional bias in PG participants with supraliminal and subliminal tasks, respectively. The dot-probe paradigm hypothesized that individuals would attribute more attentional resources to a certain emotional stimulus presented in the middle of the attended location. An advantage of this paradigm is that manipulating a stimulus onsets asynchrony. In other words, the time interval between the presentation of stimuli and the probe allows the attentional allocation at different stages of consciousness to be investigated (Bar-Haim et al., 2007). As the dot-probe task is implicit and does not require instruction and individuals need no training to complete the procedure, it’s potentially suitable for exploring and comparing different attentional processes (van Rooijen et al., 2017). van Rooijen et al. (2017) systematically and comprehensively reviewed the application of the dot-probe task since it had been developed by MacLeod et al. (1986) and summarizes factors that may potentially have an impact on the reliability and validity of dot-probe task. One of these issues is stimulus presentation duration. Most dot-probe research has used a stimulus presentation duration of 500 ms to evaluate initial reactions to the primed stimulus, and a shorter, possibly subliminal, stimulus presentation was designed in some studies (van Rooijen et al., 2017).

Focusing on threatening information is the main way to avoid harm; hence, a study on attentional bias could help us to understand the underlying mechanism of its onset and development. Maccallum and Bryant’s (2010) investigation on information processing in CG individuals using the emotional Stroop paradigm suggested the existence of an attentional bias toward loss-related events. Their study provided initial evidence of preferential processing of information related to the death of a loved one. However, the emotional Stroop task results may be interpreted in multiple ways. The results may suggest either slower attention or avoidance of all objects; thus, the results may be ambiguous. Furthermore, other researchers demonstrated that a delayed reaction to threatening stimuli may be because of other cognitive processing oriented toward an intended target (e.g., color naming) that was not related to attention (Algom et al., 2004). With the purpose of clarifying the specific processing mechanism implicated in PG, the current study adopted the dot-probe paradigm to detect subsequent reactions to specific stimuli.

In the present study, both the supraliminal and subliminal conditions involved the presentation of death- and life-related words paired with neutral words. Conducting both supraliminal and subliminal tasks may help detect whether the information processing of threatening stimuli occurred at an early, automatic stage, or at a later, conscious stage. As mentioned above, previous studies have shown multiple conflicting findings indicating that both avoidance and vigilance exist in PG individuals. Hence, we hypothesized that high-PG individuals would have had an avoidant tendency to the death-related stimuli if they reacted slower to the loss-related stimuli than low-PG group and vice versa.

## Materials and Methods

### Participants

The current study was approved by the Ethics Committee of Beijing Normal University. We recruited bereaved Chinese individuals from community-based neighborhood committees and the bulletin board system (BBS) of universities in Beijing. The participants were divided into high-PG and low-PG groups based on their scores on the Prolonged Grief Questionnaire-13 (PG-13). Participants who gave a rating of 3 or more on at least three items on the PG-13 and whose prolonged grief symptoms appear at least once a week were classified as high PG. By contrast, participants who gave a rating of 2 or less on fewer than three items on the PG-13 and whose prolonged grief symptoms appear less than once a week were classified as low PG. The final sample included 34 individuals in the high-PG group and 38 individuals in the low-PG group. All participants were 16 years of age or older, had experienced bereavement, had received at least junior middle school years of schooling, were right-handed, and had normal or corrected vision. The exclusion criteria were as follows: (1) being diagnosed with psychotic disorders, (2) receiving psychological treatment, (3) taking prescribed medication, and (4) reporting suicidal ideation.

### Measures

**Demographic and bereavement-related information**, including their age, sex, educational level, place of residence, religion, marital status, and perceived family economic status, were collected from the participants. The bereavement-related information included their relationship with the deceased, time since loss, age of the deceased, expectedness of the death, and their closeness to the deceased.

**PG-13** (Prigerson et al., 2009) is a 13-item self-report questionnaire for the diagnosis of PGD. The questionnaire assesses the individual’s experiences in the past month. This questionnaire includes 11-items rated on a 5-point scale, 1-item about duration of symptoms, and 1-item on functional impairment. Exploratory factor analysis of the Chinese version of the PG-13 revealed a one-factor structure, with one factor explaining 57.53% of the variance and factor loadings of items ranging from 0.65 to 0.78 (He et al., 2013a). The internal Cronbach’s alpha coefficient of items 1–11 in the PG-13 in this study was 0.852.

**PTSD Checklist-Civilian Version** (PCL-C; Weathers et al., 1993) is a 17-item self-report scale based on the diagnostic criteria listed in the DSM-IV. Each item was rated on a 5-point scale according to the severity of the traumatic symptom in the past month. The Chinese version of the PCL-C was reported to have good internal consistency and convergent validity (Yang et al., 2007). The internal reliability of the PCL-C in this study was 0.930.

**Zung Self-Rating Depression Scale** (SDS; Zung et al., 1965) is a 20-item self-report scale assessing the frequency of depressive symptoms in the past month. Respondents have to rate each item on a 4-point scale. The Chinese version of the SDS has good reliability and validity (Wang et al., 1999). The internal reliability of the SDS in this study was 0.832.

**Zung Self-Rating Anxiety Scale** (SAS; Zung, 1971) is a 20-item self-report scale used to assess the frequency of anxiety-related symptoms in the past month. The respondents have to rate the items on a 4-point scale. The Chinese version of the SAS has good reliability and validity (Tao and Gao, 1994). The internal reliability of the SAS in this study was 0.858.

### Experimental Design

A 2 (grief level) × 2 (consciousness level) × 3 (word type) three-factor mixed design was used in the present study. The between-subject variable was the grief level (high-PG and low-PG groups). The first within-subject variable was the consciousness level (supraliminal and subliminal levels). The second within-subject variable was the word type (life-related, death-related, and neutral two-character Chinese words). There were six words in the life-related word condition (i.e., *life, live, born, birth, pregnant*, and *breed*) and another six in the death-related word condition (i.e., *death, die, grave, gravestone, funeral*, and *bereavement*). The life-related and death-related words were, respectively, paired with 12 neutral words (e.g., *district, content, yard, industry, watch*, and *cotton*). All words were matched for frequency of use. Six additional non-target neutral words were used during the practice phase before the formal experiment (e.g., *pen, desk*, and *book*).

### Experimental Materials Check

Twenty college students (10 males, 10 females) rated the degree of emotional arousal and pleasure of the six life-related words and six death-related words used in the current study. The order of the words was randomized across the raters. One-way ANOVA revealed that the participants perceived no significant difference between the life-related and death-related words for their standardized degree of emotional arousal [*F*(11) = 0.19, *p* = 0.997] and pleasure [*F*(11) = 0.86, *p* = 0.572]. Furthermore, one-sample *t*-test indicated that the life-related words aroused positive emotions [arousal: *t*(119) = 6.61, *p* < 0.001; valence: *t*(119) = 15.65, *p* < 0.001], whereas the death-related words aroused negative emotions [arousal: *t*(119) = 4.13, *p* < 0.001; valence: *t*(119) = -12.29, *p* < 0.001].

### Dot-Probe Task

To explore whether individuals show vigilance or avoidance toward different information, we used the dot-probe paradigm (Tata et al., 1986), which has been proven to be a more sensitive paradigm when investigating competition for attention among different stimuli (Bar-Haim et al., 2007).

**FIGURE 1:**
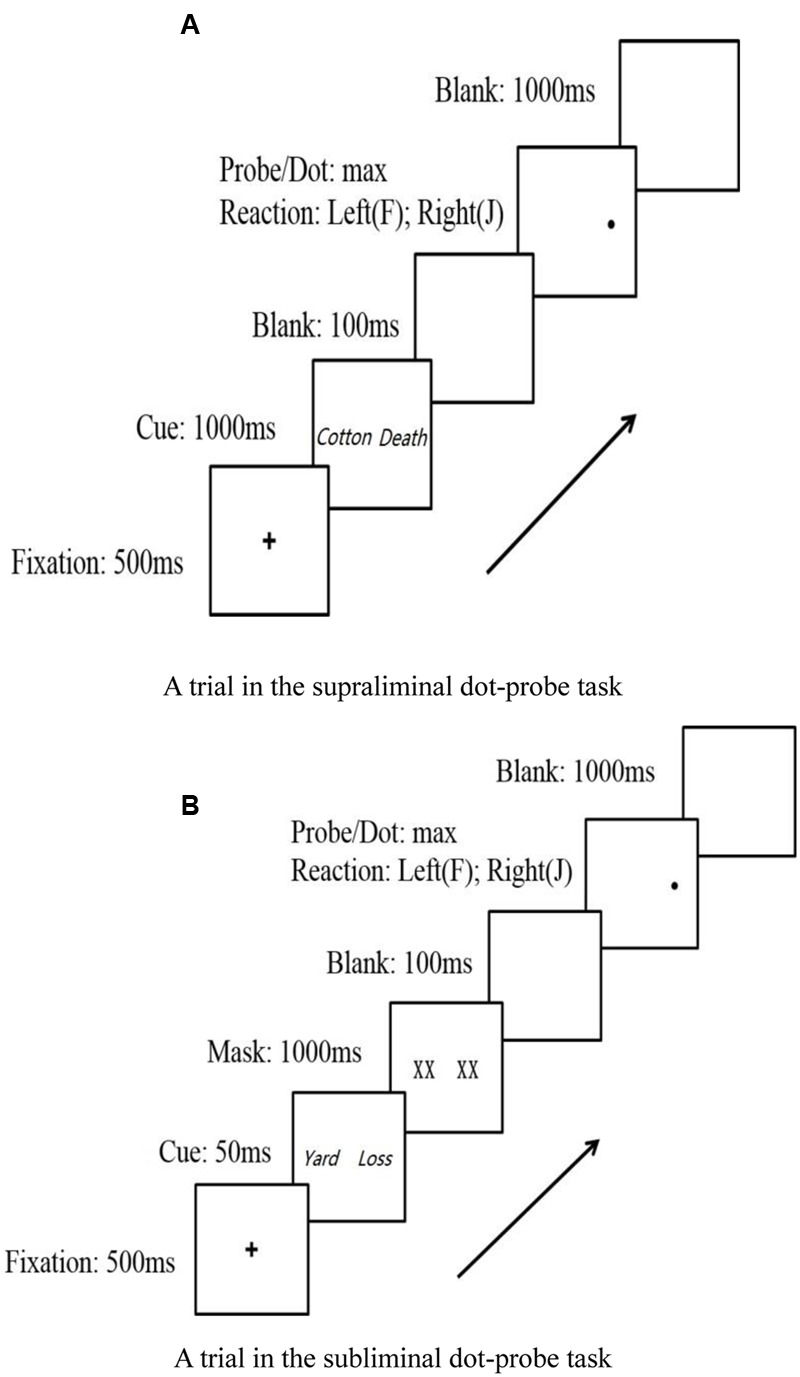
A trial in dot-probe task. **(A)** A trial in the supraliminal dot-probe task. **(B)** A trial in the subliminal dot-probe task.

**Figure 1A** shows one trial involving the supraliminal task. Initially, a fixation cross was presented to the participants; it appeared at the center of the screen for 500 ms. Then, a pairing of words (either one life-related and one neutral word or one death-related and one neutral word) was presented to participants: one word was located to the left of the fixation cross and the other to the right of the fixation cross. After an additional 1000 ms, both words disappeared, and then, a small dot appeared in the location of either word. Afterward, the participants were required to indicate whether the dot appeared on the left or right by pressing the “F” key or “J” key, respectively, on the keyboard. Participants’ reaction accuracy and time were recorded. As soon as the participants responded, the dot slide was replaced with a blank slide. After 1000 ms, the next trial began. Preferential attention was considered to occur when the participants were faster to respond to dots that replaced life-related or death-related words than to those that replaced neutral words. In contrast, when participants were slower to respond, it was considered an avoidance tendency to certain words.

**Figure 1B** shows one trial involving the subliminal task. The difference between the supraliminal and subliminal tasks was the presentation time of the cue words. In the supraliminal task, we presented the cue words for 1000 ms, and the task involved conscious processing. In the subliminal task, the cue words were presented for 50 ms, and the task detected pre-conscious bias by immediately masking the cue words with a pair of two-character words consisting of fake Chinese characters (presented for 1000 ms). This backward masking was used to interrupt sensory processing, preventing the cue words from reaching awareness (Di et al., 2000).

To reduce a practice effect, we conducted practice trials prior to the formal experiment. After each practice trial, the participants received feedback on their accuracy. The formal experiment included two tasks, and each task included 48 trials. No feedback was given during this phase. A randomized order and location of the words were presented across the trials and participants. Similarly, the order of the task type (supraliminal vs. subliminal) was randomized across the participants, who had a 5-min break between the two tasks. All the tasks were presented on notebook computers with 14-inch liquid crystal displays with E-Prime software.

### Procedure

After obtaining informed consent from the participants, the researchers asked them to complete the questionnaire package, including the background information questionnaire, PG-13, PCL-C, SDS, and SAS. Next, the participants took part in the two experiments, which involved measuring their reaction time to different stimuli. The entire formal process for each participant lasted approximately 20 min. Each participant received 20 Chinese *yuan* for their participation after completing all procedures. We also provided the participants with a booklet on grief recovery and the phone number of the campus counseling office where the researchers worked.

### Data Analysis

Participants whose accuracy rates were less than 90% or whose reaction time deviated by more than three standard deviations from their own mean reaction time were to be excluded before the statistical analysis. However, because no participant data needed to be excluded, there were 72 final valid cases. A bias index was computed based on the reaction times for each word type according to the following formula that MacLeod et al. (1986) proposed for the dot-probe paradigm: bias index = ((eldr - eldl) + (erdl - erdr))/2, where e, emotional word; d, dot; l, left position; and r, right position. A positive bias index indicates a selective attentional bias toward the location of the emotional word (i.e., vigilance), whereas a negative score indicates a bias away from the emotional word (i.e., avoidance).

SPSS 16.0 was used to calculate the reaction times and bias indexes for the correct trials. We conducted repeated measures analysis, simple effect analysis, and one-sample *t*-tests to examine the differences between the two PG groups, two word types, and two consciousness levels, as well as the interactions among all of them. Then, regression analysis using the backward deletion procedure was conducted to examine the effect of predictors accounting for the difference in reaction times (i.e., the bias index).

## Results

### Descriptive Analyses

**Table 1** presents the participants’ demographic information, bereavement-related information, and the severity of symptoms. The demographic data indicate that there were significant differences between the high-PG group and low-PG group regarding place of residence and educational level. In the current study, place of residence and educational level were dichotomous variables that were classified as coming from rural or urban regions and being educated beyond high school or not (see **Table 1**). In the high-PG group, the deceased were younger than those in the low-PG group. Moreover, the participants in the high-PG group scored higher on the PG-13, PCL-C, SDS, and SAS than those in the low-PG group.

**Table 1:** Demographic variables.

	High PG *n* = 34	Low PG *n* = 38	χ^2^/*t*
**Demographic variables**			
Age	28.63 (10.6)	29.00 (9.0)	0.17
% of females	64.7% (22)	65.8% (25)	0.01
% of urban residents	64.7% (22)	86.8% (33)	4.88^∗^
% of those unmarried	64.7% (22)	57.9% (22)	0.35
% of those educated beyond high school	79.4% (27)	100% (38)	8.67^∗∗^
% of those having no religion	82.4% (28)	94.7% (36)	2.79
Perceived family economic status^a^	3.00 (0.4)	2.92 (0.4)	0.85
**Bereavement-related variables**			
% of parents/grandparents/siblings	73.6% (25)	76.3% (29)	0.07
% of unexpected deaths	44.1% (15)	48.4% (15)	0.16
Post-loss months	46.1 (57.7)	63.6 (64.2)	1.21
Age of the deceased	60.5 (21.4)	70.2 (15.8)	-2.21^∗^
Closeness to the deceased^b^	4.50 (0.8)	4.16 (0.9)	1.72
**Severity of symptoms**			
Prolonged grief (PG-13)	23.9 (4.6)	14.3 (2.2)	11.60^∗∗∗^
PTSD (PCL-C)	34.6 (11.4)	24.6 (6.6)	4.62^∗∗∗^
Depression (SDS)	38.9 (7.5)	33.4 (6.5)	3.34^∗∗^
Anxiety (SAS)	35.2 (7.1)	29.8 (6.6)	3.33^∗∗^

*Standard deviation or the relevant number appears in parentheses.*

*^a^5-point scale on perceived family economic status, ranging from very poor (1) to very wealthy (5).*

*^b^5-point scale on closeness to the deceased, ranging from very distant (1) to very close (5).*

*^∗^*p* < 0.05; ^∗∗^*p* < 0.01; ^∗∗∗^*p* < 0.001.*

### Attentional Bias

**FIGURE 2:**
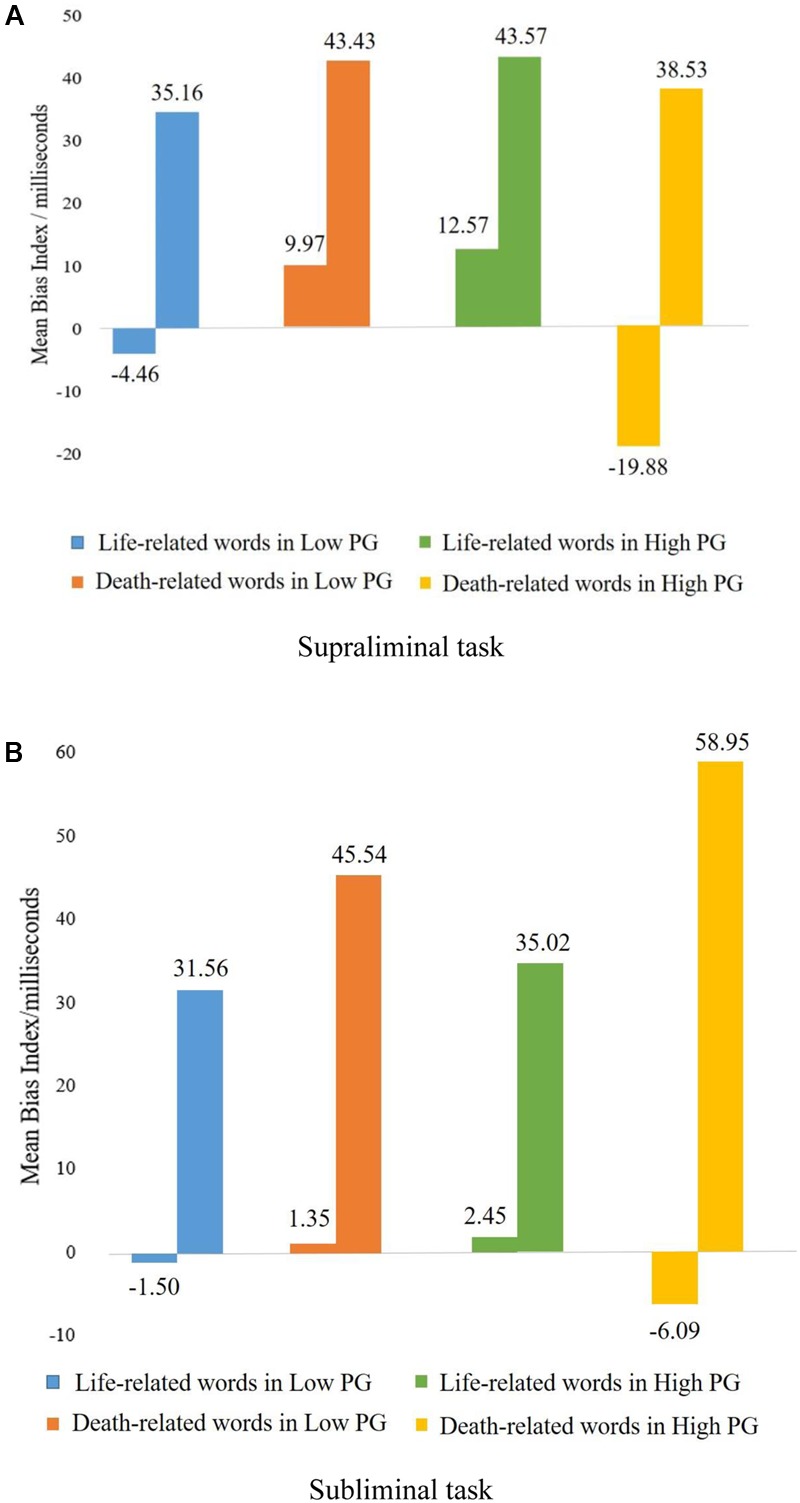
Mean bias indexes and SE (milliseconds) in the dot-probe task. **(A)** Supraliminal task. **(B)** Subliminal task.

**Figures 2A,B** present the average bias indexes and standard error for the life-related/death-related words for the supraliminal and subliminal task conditions for the high-PG group and low-PG group.

Since a bias index was used in the current study, the number of word types was changed from 3 (death-related, life-related, and neutral) to 2 (death-related bias and life-related bias) in the process of computation. The 2 (prolonged grief level) × 2 (consciousness level) × 2 (word type: death-related bias and life-related bias) mixed model ANOVA analysis indicated no main effect for the grief level [*F*(1,69) = 0.83, *p* = 0.367], consciousness level [*F*(1,69) = 0.03, *p* = 0.871], or word type [*F*(1,69) = 1.22, *p* = 0.273]. However, a significant interaction effect was found between the grief level and word type [*F*(1,69) = 8.12, *p* = 0.006, $\eta_{p}^{2}$ = 0.11]. To further examine the interaction effect, a simple effect test was conducted. The results showed a significant simple effect of word type in the high-PG group [*F*(1,138) = 6.63, *p* = 0.011] and a significant simple effect of the grief level on death-related bias [*F*(1,138) = 7.61, *p* = 0.007]. These findings indicated that high-PG participants tended to avoid death-related words in both supraliminal and subliminal tasks. Furthermore, independent sample *t*-tests between low- and high-PG groups on four bias indexes revealed a significant difference in only reacting to death-related words in the supraliminal task (*t* = 3.07, *p* < 0.01), suggesting a tendency to be vigilant toward stimuli related to death in the low-PG group.

As the high-PG and low-PG groups also differed in terms of some background information and the severity of other symptoms, the pattern of attentional bias may have also been influenced by these variables. Regression analysis was conducted to examine this possibility. Place of residence, educational level, and PG-13, PCL-C, SDS, and SAS scores were selected as predictors, and the bias index of death-related words was entered as the dependent variable. The probability of *F* to remove a predictor was set at *p* > 0.10.

For the supraliminal dot-probe task, place of residence and SDS score were removed from the equation in steps 2 and 3, and educational level and PCL-C score were removed in steps 4 and 5, with no significant change in the amount of variance predicted by the equation. Then, the SAS score was removed from the equation. Eventually, the PG-13 score emerged as a significant predictor in the final equation [*F*(21,70) = 5.03, *p* = 0.028]. The same procedure was conducted for the data from the subliminal task. The score on SAS was the only significant predictor in the final equation [*F*(1,69) = 8.62, *p* = 0.005; see **Table 2**].

**Table 2:** Summary of final backward regression model for death-related bias.

	Variable	*B*	*SE B*	*β*	*t*	*P*
Supraliminal task^a^	PG-13	-1.18	0.84	-0.26	-2.24	0.028
Subliminal task^b^	SAS	-2.38	0.81	-0.33	-2.94	0.005

*^a^*R*^*2*^ = 0.083, adjusted *R*^*2*^ = 0.057.*

*^b^*R*^*2*^ = 0.163, adjusted *R*^*2*^ = 0.152.*

## Discussion

The primary objective of the current study was to investigate the information-processing biases associated with supraliminal/subliminal task assignment among bereaved Chinese individuals using a dot-probe paradigm. In the supraliminal and subliminal tasks, high-PG individuals reacted slower to death-related words than to life-related words, suggesting a tendency to divert their attention away from death-related words, which is largely in line with previous research (Prigerson et al., 1999; Shear et al., 2007; Boelen and Bout, 2010; Bullock and Bonanno, 2013). Low-PG participants did not avoid death-related words but showed a tendency to be consciously vigilant to death-related stimuli. The absolute value of the bias index of high-PG individuals toward threat-related stimuli was larger in subliminal tasks than in supraliminal tasks, suggesting that when consciousness is involved in the cognitive process, participants tend to suppress their more natural and automatic responses.

This study found that high-PG bereaved individuals turned away from death-related stimuli, unconsciously driven by anxiety, and showed avoidance toward negative words, consciously induced by grief. These findings suggested that PG individuals struggle to address their negative emotion. The inability to confront the sadness may have led them to avoid reminders related to the loss (Bullock and Bonanno, 2013). Specifically, when primed with negative stimuli subliminally, the fear of confronting the reality of the loss may lead to avoidance, which is an automatic and unintended form of processing. By contrast, in the supraliminal task, PG individuals exhibited an avoidance toward threat-related information triggered more by prolonged grief. It is possible that grief results from more complicated processing, including anxiety, catastrophic thinking about the grief reaction, and an inability to integrate this reality into pre-existing memory. (Boelen and Bout, 2010).

Dot-probe task results showed that an avoidance tendency existed in both supraliminal and subliminal tasks. To further examine whether this avoidant inclination was a function of PG symptoms, a backward deletion regression was conducted. In the supraliminal task, only PG-13 scores significantly predicted avoidance. This result demonstrated an avoidant tendency in high-PG individuals, which was activated by loss-related words. This result was consistent with findings of Bullock and Bonanno (2013), who discovered that individuals experiencing CG would divert their attention away from negative stimuli when primed with their deceased spouse’s name. This tendency especially emphasized the avoidance and object-specific nature of CG. Furthermore, a cognitive-behavioral conceptualization model about grief by Boelen et al. (2006) proposed that avoiding loss-related stimuli and activities facilitating adjustment plays a vital role in the development and maintenance of PGD. The bereaved Chinese individuals in the current study demonstrated an avoidant inclination to the information associated with the loss.

Nevertheless, in the subliminal task, after backward deletion regression and partialling out other symptoms, avoidance was significantly triggered by anxiety symptoms other than PG symptoms. It is possible that there is a high comorbidity between anxiety and PG symptoms in the bereaved. For instance, Li and Prigerson (2016) reported that 53.7% of those individuals who met the criteria of PGD had significantly higher symptoms of anxiety, emphasizing the high co-morbidity of pathological grief and anxious symptoms. Mogg et al. (1995) investigated this possibility through the manipulation of a probe detection task and found that anxious participants shifted their attention toward negative words that were subliminally presented. Furthermore, in Boelen and colleagues’ model (Boelen et al., 2006), it is also hypothesized that the mourners adopting anxious avoidance strategies were afraid of confronting the reality of the loss, believing that the confrontation would lead to losing control or other unbearable consequences (Boelen et al., 2006). Another explanation may be that avoiding stimuli associated with the loss may protect them from the fear of confronting these reminders (Boelen and Eisma, 2015). It is also possible that avoidance and anxiety have a mutual relationship or that avoidance may mediate the relationship between anxiety and PG symptoms. Future research is needed to further elucidate the relationships among anxiety, avoidance, and PG symptoms in a larger bereaved population.

As far back as the last century, Freud (1917/1957) proposed that bereaved persons should counter avoidance by engaging in “grief work” to address the death of a loved one. Boelen et al. (2006) also indicated that bereaved individuals may avoid situations, places, and objects after the loss of a near and dear one. Although this study demonstrated that PG participants avoided negative stimuli, some studies noted that perhaps PG individuals primarily focused on memories of the deceased and were preoccupied with positive emotions, thoughts, and memories about “how it used to be” (Shear et al., 2005). Consequently, in the future, psychotherapists may pay more attention to psychoeducation and normalization, which may help PG patients accept their negative reactions and the fact of their loss. Acceptance and Commitment Therapy (ACT), a therapeutic approach focused on emotional avoidance and subsequent behavioral change (Hayes and Wilson, 1994), may be a potential therapy targeting experiential avoidance for PG or even PGD individuals.

In addition to the main findings, group differences in demographic and bereavement-related information confirmed features of high-PG individuals who were mentioned in previous research (He et al., 2013b, 2014; Xu et al., 2015). Moreover, the high-PG group scored higher on SAS, SDS, and PCL-C, suggesting higher levels of anxiety, depression, and PTSD symptoms, except PG symptoms. Several studies have indicated that PGD is often comorbid with anxiety, depression, and PTSD (Boelen and Prigerson, 2007; Kersting et al., 2007; He et al., 2014). Therefore, it is consistent with previous literature that the high-PG participants reported higher scores on the other three symptoms. In addition, group differences in PTSD symptoms inform the understanding of the distinction between PTSD and PGD, suggesting a possibility of comorbid post-traumatic stress symptoms in the bereaved. Previous studies have shown that PG individuals experience post-traumatic stress symptoms after bereavement (Zisook et al., 1998; Shang, 2016). As there was no cut-off value in Chinese version of the PCL-C in the present study, we did not know whether high-PG participants scoring high in PCL-C also met the diagnosis of PTSD. To exclude the influence of PTSD on participants’ reaction tendency, PCL-C was controlled for in later regression analyses. However, the results demonstrated that PCL-C did not show a significant change in the amount of the variance predicting the bias index in the supraliminal task or in the subliminal task. Therefore, avoidance was induced more by grief rather than by PTSD.

The current study does have certain limitations. First, Bar-Haim et al. (2007) noted that the valence-related effects observed with words may reflect high familiarity and subjective frequency when using threat words rather than a threat-related attentional bias. To address this limitation and improve the presentation of the words, previous research has suggested that before conducting a formal experiment, researchers should gather more information about what participants fear or feel threatened by and to what degree; this information should then be integrated into the experimental design (Leutgeb et al., 2015). Hence, using pictures or human faces as pictorial stimuli in recent studies may result in more spontaneous reactions in the tasks of the current study (Fox et al., 2001; Bar-Haim et al., 2007; Miyazaki et al., 2012; Eisma et al., 2015).

In addition, there were a total of three demographic variables, educational level, place of residence and the age of the deceased, that differed among the groups, which may have contributed to a bias in the development of PG symptoms. Future studies should be designed to match participants’ background information across groups to control potential confounding factors in explaining group differences. It is important to note that although we divided the PG-13 score into a high scoring group and a low scoring group, we did not use the PGD (Prigerson et al., 2009) diagnosis because too few subjects met the PGD criteria. Thus, the high-PG group was a subclinical sample, and the results cannot be generalized to clinical situations. Future research is needed to replicate these findings in a group of subjects who meet diagnostic criteria for PGD. Nonetheless, the findings from the subthreshold cases examined in this study suggested that high levels of avoidance associated with PGD symptom severity may serve as a conservative estimate and are likely to be amplified in a sample that included diagnosed cases of PGD.

Despite these limitations, the current study did provide initial evidence that high-PG participants showed an inclination to avoid death-related information at the stage of conscious information processing. More importantly, to our knowledge, this study was the first to use an experimental design to examine vigilance or avoidance in Chinese individuals with PG symptoms. Furthermore, the current study may serve to enhance our understanding of the pathological mechanisms underlying PG, which may inform psychological interventions with PG patients, such as interventions involving the use of cognitive techniques.

## Ethics Statement

This study was carried out in accordance with Ethics Committee of Beijing Normal University with written informed consent from all subjects. All subjects gave written informed consent in accordance with the Declaration of Helsinki. The protocol was approved by the Ethics Committee of Beijing Normal University.

## Author Contributions

MY mainly contributed to the data analysis and manuscript writing. ST mainly contributed to the research design and data collection. CW, ZX, and WY assisted to recruit the participants and complete the experiment. WX contributed his knowledge to the logic of manuscript. HP contributed her intelligence and experience to the final discussion and language revision of the manuscript. Finally, JW was in charge of and supervised the whole process of research.

## Conflict of Interest Statement

The authors declare that the research was conducted in the absence of any commercial or financial relationships that could be construed as a potential conflict of interest.
